# Value of Contrast-Enhanced Ultrasound in the Preoperative Evaluation of Papillary Thyroid Carcinoma Invasiveness

**DOI:** 10.3389/fonc.2021.795302

**Published:** 2022-01-14

**Authors:** Lei Chen, Luzeng Chen, Zhenwei Liang, Yuhong Shao, Xiuming Sun, Jinghua Liu

**Affiliations:** Department of Ultrasound, Peking University First Hospital, Beijing, China

**Keywords:** papillary thyroid carcinoma, extracapsular extension, lymph node metastasis, contrast-enhanced ultrasound, invasiveness

## Abstract

**Objective:**

To evaluate the diagnostic performance of preoperative contrast-enhanced ultrasound (CEUS) in the detection of extracapsular extension (ECE) and cervical lymph node metastasis (LNM) of papillary thyroid carcinoma (PTC) and the added value of CEUS in the evaluation of PTC invasiveness to conventional ultrasound (US).

**Materials and Methods:**

A total of 62 patients were enrolled retrospectively, including 30 patients with invasive PTCs (Group A, ECE or LNM present) and 32 patients with non-invasive PTCs (Group B). All patients underwent US and CEUS examinations before surgery. US and CEUS features of PTCs and lymph nodes were compared between groups. Sensitivity, specificity, positive predictive value (PPV), negative predictive value (NPV), and accuracy of US, CEUS, and the combination of the two in the detection of ECE and LNM of PTCs were calculated. Logistic regression was used to analyze relationships between variables.

**Results:**

The PTC size was larger in group A on both US and CEUS (*P* = 0.001, *P* = 0.003). More PTCs showed hyper-enhancement in group A (*P* = 0.013) than in group B. More PTCs had >25% contact between PTC and the thyroid capsule and discontinued capsule on US and CEUS (all *P* < 0.05) in group A than in group B. More absent hilum and calcification of lymph nodes were observed in group A (both *P* < 0.05) than in group B on US. More centripetal perfusion and enlarged lymph nodes were observed in group A (both *P* < 0.05) than in group B on CEUS. CEUS alone and US combined with CEUS manifested higher diagnostic accuracy (79.0%) than US alone (72.6%) in the detection of ECE. The combination of US and CEUS manifested the highest diagnostic accuracy (95.2%) than CEUS alone (90.3%) and US alone (82.2%) in the detection of LNM. Diagnoses of ECE and LNM by the combination of US and CEUS were independent risk factors for PTC invasiveness [odds ratio (OR) = 29.49 and 97.20, respectively; both *P* = 0.001].

**Conclusion:**

CEUS or US combined with CEUS is recommended for the detection of PTC ECE, while the combination of US and CEUS is most recommended for LNM detection. CEUS plays an essential role in the preoperative evaluation of PTC invasiveness.

## Introduction

Papillary thyroid carcinoma (PTC) accounts for approximately 80%–90% of thyroid cancers (1, 2). As the incidence of PTC is gradually increasing, overtreatment of low-risk disease has become a worldwide concern (1). Although PTC is considered a moderate subtype of thyroid cancer with a preferable prognosis (3), the presence of extracapsular extension (ECE) and lymph node metastasis (LNM) are adverse prognostic factors, associated with higher recurrence and mortality rate (4). The staging and therapy plan of PTC also vary according to the above two factors. Therefore, accurate preoperative diagnosis of ECE and LNM is essential for the stratified management and precise treatment of PTC.

Among all medical imaging tools, ultrasound (US) is the first-line choice for thyroid disease assessment. Several guidelines (5–7) have been developed to improve the diagnostic performance of US for thyroid cancer, as well as new modalities such as elastography (8, 9). However, US was found to be a useful tool in the evaluation of PTC invasiveness with certain limitations. The reported accuracy for ECE detection by US was around 64.7%–92.6% (10, 11), while the reported accuracy for LNM detection was around 51.9%–84.3% (12, 13). To generalize the use of US in PTC invasiveness assessment in clinical settings, improvement of diagnostic performance is required.

Contrast-enhanced ultrasound (CEUS) techniques have rapidly advanced in the diagnosis of endocrinological tumors, including thyroid cancer, testicular neoplasm, and paraganglioma (14–16). Superior in the detection of microvascularity of tissue, CEUS was proven to be a promising tool in the evaluation of ECE and LNM of PTC (17–23). However, most of the studies focused on either ECE or LNM of PTC. Comprehensive analysis of PTC invasiveness lacked. In addition, inconsistency was found among different studies.

In this study, we investigated the US and CEUS features of invasive PTC, analyzed the diagnostic performance of US and CEUS in the detection of ECE and LNM, and explored the relationship between imaging features and PTC invasiveness, aiming to verify the added value of CEUS in the evaluation of PTC invasiveness.

## Materials and Methods

### Patients and Study Design

This study was approved by the ethics committee of our hospital. All patients signed an informed consent before examination. From February 2017 to December 2017, a total of 190 patients who underwent US and CEUS examinations of thyroid and cervical lymph nodes in our hospital were reviewed.

Inclusion criteria were as follows: 1) Surgical pathology-approved PTC; 2) US and CEUS examination within 1 month before surgery; 3) PTC size ≥0.5 cm; 4) Lymph node long-axis diameter + short-axis diameter ≥1.0 cm.

Exclusion criteria were as follows: 1) Patients with incomplete data; 2) Unsatisfied demonstration of PTC boundary; 3) Pathology-approved PTC could not be identified on US.

The presence of ECE and LNM was confirmed by surgical pathology. ECE was defined as positive if there was minimal microscopic extrathyroidal extension to surrounding soft tissues, strap muscles, or gross extrathyroidal extension to nerves, blood vessels, and other neighboring organs on pathology. Meanwhile, lymph nodes were considered benign if 1) Diagnosed as benign by surgical pathology or core needle biopsy (CNB); 2) There was a <20% increase in size and absence of US features suspicious for malignancy after a 2-year follow-up (18).

Finally, 62 patients were enrolled, including 51 females and 11 males, with an average age of 45.8 ± 13.4 years.

### Ultrasound Examination

All conventional US and CEUS examinations were performed by three physicians with more than 9 years of experience in ultrasound diagnosis using a GE Logiq E9 (General Electric Healthcare, Waukesha, WI, USA) ultrasonic system equipped with a 6–15 MHz linear transducer according to a standard protocol in our department, which was consistent during the study period.

All conventional US images were reviewed and confirmed by two physicians with more than 5 years of experience, without knowing the histological information. Thyroid nodule features including size, the range of contact between PTC and thyroid capsule, and the presence of discontinued capsule were recorded. Nodule size was recorded as the maximum dimension in all planes. The range of contact between PTC and the thyroid capsule was divided into no contact (normal thyroid parenchyma existed between PTC and the thyroid capsule), <25% contact, 25%–50% contact, and >50% contact based on the ratio of contact part to the whole nodule perimeter. Discontinued capsule was recorded if there was the loss of normal thyroid capsule linear echo. The nodule nearest the thyroid capsule was enrolled in multifocal lesions.

For cervical lymph nodes, features including size, shape [long-axis diameter/short-axis diameter ratio (L/S ratio)], margin (well-defined/ill-defined), echogenicity (hypoechoic, isoechoic, or hyperechoic with respect to adjacent muscles), calcification (present/absent), and blood flow distribution (avascular/hilar/peripheral/mixed type) of cervical lymph nodes were recorded (21). In case the patient was present with several suspicious lymph nodes, the largest lymph node was enrolled. If no suspicious lymph node was present, the largest one of all non-suspicious lymph nodes was enrolled.

### Contrast-Enhanced Ultrasound Examination

The mechanical index (MI = 0.08–0.10) was selected automatically by the ultrasonic system in relation to beam-focus depth. SonoVue (Bracco, Milan, Italy) was used as the ultrasound contrast agent. Here, 5.0 ml solution of 0.9% saline and SonoVue were mixed by oscillation. Then, 1.2 ml SonoVue was injected as a bolus followed immediately by 5.0 ml 0.9% saline flush *via* the cephalic vein. In this study, 90 s of CEUS was recorded in real time. TomTec workstation (TOMTEC Imaging Systems GmbH, Unterschleissheim, Germany) was used for CEUS off-line analysis.

All CEUS images were reviewed and confirmed by two physicians with more than 10 years of experience without knowing the histological information.

For PTC, the size of the nodule, the degree of enhancement, the range of contact between PTC and thyroid capsule, and the presence of discontinued capsule were recorded. The degree of enhancement was divided into hyper-enhancement, iso-enhancement, and hypo-enhancement with respect to the surrounding normal thyroid parenchyma. The evaluation of the range of contact between PTC and thyroid capsule was similar to the evaluation on US. Discontinued capsule was noted when the thyroid capsule was discontinued in the early artery phase (17).

For cervical lymph nodes, enhancement direction (centripetal/centrifugal), enhancement type (no enhancement/homogeneous enhancement/peripheral enhancement/mixed enhancement), and enhancement range (enlarged or not) were recorded (21).

### Diagnostic Criteria for Extracapsular Extension and Lymph Node Metastasis

If the contact range between PTC and the thyroid capsule was >25%, and discontinued thyroid capsule was observed on US or CEUS, ECE would be diagnosed respectively (20). When combining US and CEUS together, if three or more of the above four features (>25% contact on US, discontinued capsule on US, >25% contact on CEUS, discontinued capsule on CEUS) were observed, ECE would be diagnosed by US+CEUS.

If two or more of the following features (L/S ratio <2, ill-defined margin, hyper-echogenicity, absent hilum, calcification, peripheral or mixed vascularity) were observed on US, LNM would be diagnosed by US. If two or more of the following features (centripetal perfusion, peripheral or mixed enhancement, and enlarged size on CEUS compared to US) were found on CEUS, LNM would be diagnosed by CEUS. Combining US and CEUS together, if three or more of all the above features were observed, LNM would be diagnosed by US+CEUS (21).

### Statistical Analysis

SPSS 16.0 software (IBM, Armonk, NY, USA) was used for statistical analysis. Continuous data of normal distribution were described by mean ± standard deviation, continuous data of non-normal distribution were described by median (interquartile range), and categorized data were described by percentage. Using independent-sample *t* test for the comparison of continuous data of normal distribution, Mann–Whitney test for continuous data of non-normal distribution, and chi-square test or Fisher’s exact test for the proportion comparison of categorized data. Sensitivity, specificity, positive predictive value (PPV), negative predictive value (NPV), and overall accuracy of US and CEUS in the diagnosis of ECE and LNM were calculated. Logistic regression was used to explore the relationship between variables. *P* < 0.05 (two-tailed) was considered to be statistically significant.

## Results

### Patients

Most enrolled patients were symptomless, with thyroid lesions accidentally discovered during physical examinations (49/62, 79.0%). The most common clinical symptom was painless neck mass (8/62, 12.9%), followed by hoarseness (4/62, 6.5%), cough (3/62, 4.8%), and palpitation (3/62, 4.8%). Among 59 patients (95.2%) with thyroid function test results, one had hyperthyroidism (1/62, 1.6%), while the others had normal thyroid function.

Based on pathology, 15 patients had multifocal PTCs (24.2%). Here, 16 patients had PTCs with ECE (25.8%), 11 patients had PTCs with LNM (17.7%), and 3 patients had PTCs with both ECE and LNM (4.8%). These 30 patients were group A (48.4%). The other 32 patients had PTCs without ECE or LNM (51.6%, group B). Among all patients with ECE, 3 presented with gross extrathyroidal extension (18.8%), and the others presented minimal extrathyroidal extension (13/16, 81.2%). No significant difference was found in age, gender, or multifocality between the two groups (all *P* > 0.05).

### Ultrasound Examination

Most PTCs were solid (60/62, 96.8%), hypoechoic (54/62, 87.1%) nodules with microcalcifications (31/62, 50.0%) and lobulated or irregular margins (27/62, 43.5%). In addition, 18 PTCs (29.0%) showed taller-than-wide shape. Most PTCs (50/62, 80.6%) were grade 5 based on the 2017 American College of Radiology (ACR) Thyroid Imaging, Reporting and Data System (TI-RADS) (5), while the others were grade 4.

The average size of PTC on US was 0.95 (0.68, 1.30) cm, ranging from 0.5 to 6.8 cm. PTC size of group A was significantly larger than that of group B (*P* = 0.001). More PTCs showed >25% contact with the thyroid capsule and discontinued thyroid capsule in group A than in group B (*P* < 0.001, *P* = 0.007, respectively). More absent hilum and calcification were observed in lymph nodes in group A (*P* = 0.02, *P* = 0.01, respectively), while no significant difference was found in L/S ratio, ill-defined margin, hyper-echogenicity, or vascularity distribution of lymph nodes between groups, as shown in **Table 1**.

**Table 1:** US and CEUS features of PTCs and lymph nodes in the two groups.

		Group A (n = 30)	Group B (n = 32)	*P*
US	PTC size, cm	1.20 (0.88,1.45)	0.80 (0.60,1.00)	0.001*
	Contact between PTC and thyroid capsule, n (%)			<0.001*
	>25%	19 (63.3%)	4 (12.5%)	
	≤25%	11 (36.7%)	28 (87.5%)	
	Presence of discontinued capsule, n (%)			0.007*
	Yes	16 (53.3%)	6 (18.8%)	
	No	14 (46.7%)	26 (81.2%)	
	LN L/S ratio, n (%)			0.103
	≥2	17 (56.7%)	25 (78.1%)	
	<2	13 (43.3%)	7 (21.9%)	
	Margin of LN, n (%)			0.077
	Well-defined	23 (76.7%)	30 (93.8%)	
	Ill-defined	7 (23.3%)	2 (6.2%)	
	Hyper-echogenicity in LN, n (%)			0.099
	Present	25 (83.3%)	31 (96.9%)	
	Absent	5 (16.7%)	1 (3.1%)	
	Hilum structure, n (%)			0.020*
	Present	18 (60.0%)	28 (87.5%)	
	Absent	12 (40.0%)	4 (12.5%)	
	Calcification in LN, n (%)			0.010*
	Present	6 (20.0%)	0 (0.0%)	
	Absent	24 (80.0%)	32 (100%)	
	Peripheral or mixed blood flow, n (%)			0.249
	Present	5 (16.7%)	2 (6.2%)	
	Absent	25 (83.3%)	30 (93.8%)	
CEUS	PTC size, cm	1.15 (0.70, 1.45)	0.70 (0.50, 0.98)	0.003*
	Degree of enhancement, n (%)			0.013*
	Hyper-enhancement	6 (20.0%)	0 (0.0%)	
	Iso-enhancement	1 (3.3%)	2 (6.2%)	
	Hypo-enhancement	23 (76.7%)	30 (93.8%)	
	Contact between PTC and thyroid capsule, n (%)			<0.001*
	>25%	21 (70.0%)	3 (9.4%)	
	≤25%	9 (30.0%)	29 (90.6%)	
	Presence of discontinued capsule, n (%)			<0.001*
	Yes	17 (56.7%)	4 (12.5%)	
	No	13 (43.3%)	28 (87.5%)	
	Perfusion direction of LN, n (%)			<0.001*
	Centripetal	11 (36.7%)	0 (0.0%)	
	Centrifugal	19 (63.3%)	32 (100%)	
	Peripheral or mixed enhancement of LN, n (%)			0.125
	Present	17 (56.7%)	11 (34.4%)	
	Absent	13 (43.3%)	21 (65.6%)	
	Enlarged range on CEUS, n (%)			0.029*
	Yes	10 (33.3%)	3 (9.4%)	
	No	20 (66.7%)	29 (90.6%)	

LN, lymph node; PTC, papillary thyroid carcinoma; US, ultrasound; CEUS, contrast-enhanced ultrasound.

*Statistically significant.

### Contrast-Enhanced Ultrasound Examination

The average size of PTC on CEUS was 0.85 (0.60, 1.20) cm. As shown in **Table 1**, PTC size of group A was significantly larger than that of group B (*P* = 0.003). More PTCs with hyper-enhancement compared to the surrounding thyroid parenchyma were observed in group A, while more PTCs with hypo-enhancement were observed in group B (*P* = 0.013). More PTCs demonstrated >25% contact with the thyroid capsule and discontinued thyroid capsule in group A (*P* < 0.001). More centripetal perfusion and enlarged size of LNs were observed in lymph nodes in group A (*P* < 0.001, *P* = 0.029, respectively), while no significant difference was found in the enhancement type of LNs (*P* > 0.05).

### Diagnostic Performance Analysis

**Figure 1:**
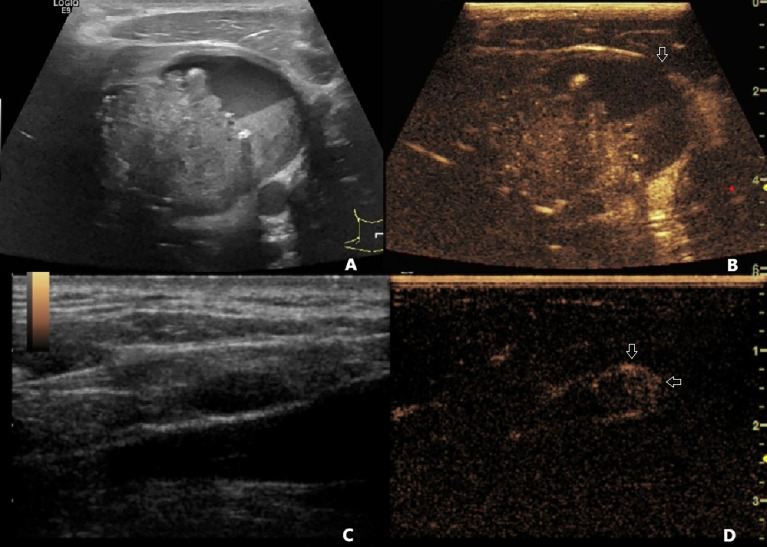
**(A)** A 40-year-old male patient with pathology-confirmed PTC underwent preoperative US and CEUS examination. **(A)** One 6.8 cm * 4.7 cm mixed cystic and solid nodule was found in the left lobe of the thyroid. The contact range between PTC and the thyroid capsule was >50%. The capsule was continued on US. **(B)** On CEUS, the contact range between PTC and the thyroid capsule was >50%, and discontinued capsule was observed (white arrow). **(C)** A 1.2 cm * 0.5 cm hypoechoic lymph node with clear boundary and unclear hilum structure was found on US. **(D)** This lymph node demonstrated centripetal perfusion and peripheral enhancement on CEUS (white arrows). This patient was diagnosed with non-invasive PTC by US, PTC with ECE and LNM by CEUS and US+CEUS, and was finally confirmed to have PTC with ECE and LNM by pathology. PTC, papillary thyroid carcinoma; US, ultrasound; CEUS, contrast-enhanced ultrasound; ECE, extracapsular extension; LNM, lymph node metastasis.

CEUS alone and the combination of US and CEUS were more accurate than US alone in the diagnosis of ECE of PTC. However, the combination of US and CEUS did not make much improvement in the diagnostic accuracy than CEUS alone. Meanwhile, the combination of US and CEUS was more accurate than US or CEUS alone in the diagnosis of LNM from PTC. CEUS alone was more accurate than US alone as well. The results are shown in **Tables 2**, **3** and **Figure 1**.

**Table 2:** Diagnostic performance of US, CEUS, and US combined CEUS for ECE of PTC.

Modalities	Sensitivity (%)	Specificity (%)	PPV (%)	NPV (%)	Accuracy (%)
US	52.6	81.4	55.6	79.5	72.6
CEUS	68.4	83.7	65.0	85.7	79.0
US+CEUS	73.6	81.4	63.6	87.5	79.0

PTC, papillary thyroid carcinoma; ECE, extracapsular extension; US, ultrasound; CEUS, contrast-enhanced ultrasound; PPV, positive predictive value; NPV, negative predictive value.

**Table 3:** Diagnostic performance of US, CEUS, and US combined CEUS for LNM from PTC.

Modalities	Sensitivity (%)	Specificity (%)	PPV (%)	NPV (%)	Accuracy (%)
US	78.5	83.3	57.9	93.0	82.2
CEUS	78.5	93.8	78.6	93.8	90.3
US+CEUS	92.9	95.8	86.7	97.9	95.2

PTC, papillary thyroid carcinoma; LNM, lymph node metastasis; US, ultrasound; CEUS, contrast-enhanced ultrasound; PPV, positive predictive value; NPV, negative predictive value.

### Logistic Regression Analysis

Based on the above statistical analysis results and clinical data, age, gender, PTC size, degree of enhancement, diagnosis of ECE by US+CEUS, and diagnosis of LNM by US+CEUS were used in binary logistic regression analysis. The results showed that the diagnoses of ECE and LNM by the combination of US and CEUS were independent risk factors for PTC invasiveness [odds ratio (OR) = 29.49, 97.20, respectively; both *P* = 0.001].

## Discussion

In this study, we found that the PTC size in group A was significantly larger than that in group B on both US and CEUS. Nodule size was reported to be an independent risk factor for differentiated thyroid cancer (22–25). This might be due to the aggressive nature of invasive PTC that the aggregation of tumor cells might be promoted at the gene or molecular level compared with non-invasive PTC (26). Meanwhile, more hyper-enhancement was observed in group A than in group B. Generally speaking, PTC tends to be hypo-enhanced compared to benign nodules of the thyroid, since they lack blood supply (27, 28), especially when the PTC size is small (29). However, we found that 20% of PTCs in group A were hyper-enhanced, even though two-thirds of them were smaller than 2.0 cm. Recently, intranodular vascularity evaluated by US has no longer been considered an independent risk factor for malignancy in PTCs (4). Nevertheless, we speculate that vascularity evaluated by CEUS might be associated with PTC invasiveness, consistent with other studies (22, 23, 30). Since angiogenesis is fundamental in the development, growth, and metastasis of PTC (23), this sign might suggest more active neovascularization and greater microvascular density in invasive PTC, which could be reflected by CEUS.

PTCs in group A had a larger contact range between PTC and the thyroid capsule and more presence of discontinued capsule than PTCs in group B. Meanwhile, lymph nodes in Group A showed more absent hilum and calcification on US, as well as more centripetal perfusion and enlarged size on CEUS than in group B. The above ultrasonic features were reported to be indicators for PTC ECE and LNM (17, 18, 21, 31), and our results consistently demonstrated its importance in PTC invasiveness evaluation. These findings helped us to obtain a better understanding of the ultrasonic characterization of invasive PTC.

Several studies have focused on the application of CEUS in the diagnosis of ECE or LNM from PTC (17–23, 28, 31). Most of them suggested that CEUS had better diagnostic performance than US. In this study, we had consistent findings. In both ECE and LNM diagnosis, CEUS showed higher sensitivity, specificity, PPV, NPV, and accuracy than those of US. CEUS is dominant in the detection of tissue perfusion. It is sensitive to microvascularity; hence, it has advantages in the display of lesion contour, thyroid capsule interruption, and hemodynamics of PTC and lymph nodes. These might be the fundamentals of the better diagnostic performance shown by CEUS. Interestingly, for ECE diagnosis, the combination of US and CEUS did not show much improvement in the diagnostic accuracy other than CEUS alone. This might be because for ECE diagnosis, the parameters obtained by US and CEUS were the same. CEUS was superior in the detection of PTC and capsule contact as well as capsule continuity (20), working more like a substitution than a supplement to US. However, for LNM diagnosis, US and CEUS showed different advantages. CEUS is more sensitive in the detection of perfusion direction and microvascularity structure than US (32), yet it has difficulty recognizing other features obtained by US, such as calcification and hyper-echogenicity. Thus, the combination of US and CEUS showed the highest accuracy, followed by CEUS and US alone.

The binary logistic regression analysis showed that the diagnoses of ECE and LNM by the combination of US and CEUS were independent risk factors for PTC invasiveness. The success of surgery for thyroid cancer hinges on thorough and accurate preoperative imaging. At present, US is recommended for the preoperative evaluation of thyroid cancer by the American Thyroid Association (ATA) (33). In commonly used guidelines for thyroid nodule management such as 2015 ATA guideline and 2017 ACR TI-RADS, PTC smaller than 1 cm without high-risk features is considered indolent, and active clinical diagnosis and treatment are not recommended (5, 33, 34). However, effective preoperative evaluation of PTC invasiveness lacks in traditional imaging methods. In our study, we found that the diagnosis of ECE and LNM by the combination of US and CEUS could significantly predict PTC invasiveness, even in subcentimeter PTCs. Thus, we state that the combination of US and CEUS could be a promising tool for the preoperative evaluation of PTC invasiveness and might benefit the optimal stratified management of PTC patients.

There are some limitations of this study: 1) This is a retrospective study with a relatively small sample size. Selection bias might exist because patients with non-invasive micro-PTC might take clinical and ultrasonic follow-up instead of surgery. Larger and prospective studies will be helpful to generalize the results in the future; 2) In this study, size thresholds were set for enrollment of PTC and LN because movement may lead to errors in measurement and difficulty existed in the CEUS feature observation for small lesions; 3) The value of CEUS in the evaluation of different degrees of ECE and different compartment lymph node metastasis will be further addressed in our future study.

## Conclusion

US and CEUS features for invasive PTC include larger size, hyper-enhancement, >25% contact between PTC and thyroid capsule, discontinued capsule, absent hilum, calcification in lymph node, centripetal perfusion, and enlarged lymph node on CEUS. CEUS has added value in the evaluation of PTC invasiveness when compared to US. CEUS or US+CEUS is recommended for the evaluation of ECE, while US+CEUS is recommended for the evaluation of LNM. CEUS is a promising tool in the preoperative assessment of PTC invasiveness, which might benefit the clinical stratified management of PTC patients.

## Data Availability Statement

The raw data used to support the findings of this study are available from the corresponding author upon request.

## Ethics Statement

The studies involving human participants were reviewed and approved by the Ethics Committee of Peking University First Hospital. The patients/participants provided their written informed consent to participate in this study.

## Author Contributions

LC conceived and designed the analysis, collected the data, contributed data or analysis tools, performed the analysis, and wrote the paper. LZC conceived and designed the analysis, collected the data, contributed data or analysis tools, and wrote the paper. ZL collected the data, contributed data or analysis tools, and wrote the paper. YS collected the data and wrote the paper. XS collected the data and wrote the paper. JL collected the data and wrote the paper. All authors contributed to the article and approved the submitted version.

## Funding

This work was supported by Research Fund of Peking University First Hospital (grant nos. 2017CR05 and 2021CR02).

## Conflict of Interest

The authors declare that the research was conducted in the absence of any commercial or financial relationships that could be construed as a potential conflict of interest.

## Publisher’s Note

All claims expressed in this article are solely those of the authors and do not necessarily represent those of their affiliated organizations, or those of the publisher, the editors and the reviewers. Any product that may be evaluated in this article, or claim that may be made by its manufacturer, is not guaranteed or endorsed by the publisher.

## References

[1] Araque KA Gubbi S Klubo-Gwiezdzinska J. Updates on the Management of Thyroid Cancer. Horm Metab Res (2020) 52(8):562–77. doi: 10.1055/a-1089-7870 PMC7415555 32040962

[2] Sherman SI. Thyroid Carcinoma. Lancet (2003) 361(9356):501–11. doi: 10.1016/s0140-6736(03)12488-9 12583960

[3] Cabanillas ME McFadden DG Durante C. Thyroid Cancer. Lancet (2016) 388(10061):2783–95. doi: 10.1016/s0140-6736(16)30172-6 27240885

[4] Haugen BR Alexander EK Bible KC Doherty GM Mandel SJ Nikiforov YE. 2015 American Thyroid Association Management Guidelines for Adult Patients With Thyroid Nodules and Differentiated Thyroid Cancer: The American Thyroid Association Guidelines Task Force on Thyroid Nodules and Differentiated Thyroid Cancer. Thyroid (2016) 26(1):1–133. doi: 10.1089/thy.2015.0020 26462967 PMC4739132

[5] Tessler FN Middleton WD Grant EG Hoang JK Berland LL Teefey SA. ACR Thyroid Imaging, Reporting and Data System (TI-RADS): White Paper of the ACR TI-RADS Committee. J Am Coll Radiol (2017) 14(5):587–95. doi: 10.1016/j.jacr.2017.01.046 28372962

[6] Shin JH Baek JH Chung J Ha EJ Kim JH Lee YH. Ultrasonography Diagnosis and Imaging-Based Management of Thyroid Nodules: Revised Korean Society of Thyroid Radiology Consensus Statement and Recommendations. Korean J Radiol (2016) 17(3):370–95. doi: 10.3348/kjr.2016.17.3.370 PMC4842857 27134526

[7] Russ G Bonnema SJ Erdogan MF Durante C Ngu R Leenhardt L. European Thyroid Association Guidelines for Ultrasound Malignancy Risk Stratification of Thyroid Nodules in Adults: The EU-TIRADS. Eur Thyroid J (2017) 6(5):225–37. doi: 10.1159/000478927 PMC5652895 29167761

[8] Celletti I Fresilli D De Vito C Bononi M Cardaccio S Cozzolino A. TIRADS, SRE and SWE in INDETERMINATE Thyroid Nodule Characterization: Which has Better Diagnostic Performance? Radiol Med (2021) 126(9):1189–200. doi: 10.1007/s11547-021-01349-5 PMC8370962 34129178

[9] Cantisani V Maceroni P D'Andrea V Patrizi G Di Segni M De Vito C. Strain Ratio Ultrasound Elastography Increases the Accuracy of Colour-Doppler Ultrasound in the Evaluation of Thy-3 Nodules. A Bi-Centre University Experience. Eur Radiol (2016) 26(5):1441–9. doi: 10.1007/s00330-015-3956-0 26337431

[10] Lee DY Kwon TK Sung MW Kim KH Hah JH. Prediction of Extrathyroidal Extension Using Ultrasonography and Computed Tomography. Int J Endocrinol (2014) 2014:351058. doi: 10.1155/2014/351058 25525431 PMC4265702

[11] Kim H Kim JA Son EJ Youk JH Chung TS Park CS. Preoperative Prediction of the Extrathyroidal Extension of Papillary Thyroid Carcinoma With Ultrasonography Versus MRI: A Retrospective Cohort Study. Int J Surg (2014) 12(5):544–8. doi: 10.1016/j.ijsu.2014.03.003 24631554

[12] Choi JS Kim J Kwak JY Kim MJ Chang HS Kim EK. Preoperative Staging of Papillary Thyroid Carcinoma: Comparison of Ultrasound Imaging and CT. AJR Am J Roentgenol (2009) 193(3):871–8. doi: 10.2214/ajr.09.2386 19696304

[13] Hwang HS Orloff LA. Efficacy of Preoperative Neck Ultrasound in the Detection of Cervical Lymph Node Metastasis From Thyroid Cancer. Laryngoscope (2011) 121(3):487–91. doi: 10.1002/lary.21227 21344423

[14] Cantisani V Bertolotto M Weskott HP Romanini L Grazhdani H Passamonti M. Growing Indications for CEUS: The Kidney, Testis, Lymph Nodes, Thyroid, Prostate, and Small Bowel. Eur J Radiol (2015) 84(9):1675–84. doi: 10.1016/j.ejrad.2015.05.008 26014102

[15] Isidori AM Pozza C Gianfrilli D Giannetta E Lemma A Pofi R. Differential Diagnosis of Nonpalpable Testicular Lesions: Qualitative and Quantitative Contrast-Enhanced US of Benign and Malignant Testicular Tumors. Radiology (2014) 273(2):606–18. doi: 10.1148/radiol.14132718 24968192

[16] Puliani G Sesti F Feola T Di Leo N Polti G Verrico M. Natural History and Management of Familial Paraganglioma Syndrome Type 1: Long-Term Data From a Large Family. J Clin Med (2020) 9(2):588. doi: 10.3390/jcm9020588 PMC7074269 32098148

[17] Ding K Cui Q Yan K Liu W Wang T. Diagnostic Value of Conventional Ultrasound and Contrast Enhanced Ultrasound in Predicting Extrathyroidal Extension of Papillary Thyroid Cancer. Chin J Ultrason (2017) 26(3):243–8. doi: 10.2147/CMAR.S299157

[18] Hong YR Luo ZY Mo GQ Wang P Ye Q Huang PT. Role of Contrast-Enhanced Ultrasound in the Pre-Operative Diagnosis of Cervical Lymph Node Metastasis in Patients With Papillary Thyroid Carcinoma. Ultrasound Med Biol (2017) 43(11):2567–75. doi: 10.1016/j.ultrasmedbio.2017.07.010 28807450

[19] Jiang W Wei HY Zhang HY Zhuo QL. Value of Contrast-Enhanced Ultrasound Combined With Elastography in Evaluating Cervical Lymph Node Metastasis in Papillary Thyroid Carcinoma. World J Clin Cases (2019) 7(1):49–57. doi: 10.12998/wjcc.v7.i1.49 30637252 PMC6327137

[20] Chen L Chen L Liu J Liang Z Wang B. Contrast-Enhanced Ultrasound and BRAF Mutation in Diagnosis of Extracapsular Extension of Papillary Thyroid Carcinoma. Chin J Med Imaging Technol (2020) 36(1):50–4. doi: 10.13929/j.issn.1003-3289.2020.01.013

[21] Chen L Chen L Liu J Wang B Zhang H. Value of Qualitative and Quantitative Contrast-Enhanced Ultrasound Analysis in Preoperative Diagnosis of Cervical Lymph Node Metastasis From Papillary Thyroid Carcinoma. J Ultrasound Med (2020) 39(1):73–81. doi: 10.1002/jum.15074 31222782

[22] Tao L Zhou W Zhan W Li W Wang Y Fan J. Preoperative Prediction of Cervical Lymph Node Metastasis in Papillary Thyroid Carcinoma *via* Conventional and Contrast-Enhanced Ultrasound. J Ultrasound Med (2020) 39(10):2071–80. doi: 10.1002/jum.15315 32352187

[23] Zhan J Zhang LH Yu Q Li CL Chen Y Wang WP. Prediction of Cervical Lymph Node Metastasis With Contrast-Enhanced Ultrasound and Association Between Presence of BRAF(V600E) and Extrathyroidal Extension in Papillary Thyroid Carcinoma. Ther Adv Med Oncol (2020) 12:1758835920942367. doi: 10.1177/1758835920942367 32843902 PMC7418479

[24] Price DL Wong RJ Randolph GW. Invasive Thyroid Cancer: Management of the Trachea and Esophagus. Otolaryngol Clin North Am (2008) 41(6):1155–1168, ix-x. doi: 10.1016/j.otc.2008.08.002 19040976 PMC2750808

[25] Zhao Y Zhang Y Liu XJ Shi BY. Prognostic Factors for Differentiated Thyroid Carcinoma and Review of the Literature. Tumori (2012) 98(2):233–7. doi: 10.1700/1088.11935 22677990

[26] Xing M. BRAF Mutation in Papillary Thyroid Cancer: Pathogenic Role, Molecular Bases, and Clinical Implications. Endocr Rev (2007) 28(7):742–62. doi: 10.1210/er.2007-0007 17940185

[27] Pang T Huang L Deng Y Wang T Chen S Gong X. Logistic Regression Analysis of Conventional Ultrasonography, Strain Elastosonography, and Contrast-Enhanced Ultrasound Characteristics for the Differentiation of Benign and Malignant Thyroid Nodules. PloS One (2017) 12(12):e0188987. doi: 10.1371/journal.pone.0188987 29228030 PMC5724846

[28] Peng Q Niu C Zhang M Peng Q Chen S. Sonographic Characteristics of Papillary Thyroid Carcinoma With Coexistent Hashimoto's Thyroiditis: Conventional Ultrasound, Acoustic Radiation Force Impulse Imaging and Contrast-Enhanced Ultrasound. Ultrasound Med Biol (2019) 45(2):471–80. doi: 10.1016/j.ultrasmedbio.2018.10.020 30528690

[29] Zheng X Zhang Y Zhao C Shi X Li C Jiang J. Diagnosis of Thyroid Space-Occupying Lesions Using Real-Time Contrast-Enhanced Ultrasonography With Sulphur Hexafluoride Microbubbles. Chin J Of Otorhinolaryngol Head And Neck Surg (2009) 44(4):5. doi: 10.1016/j.ceramint.2007.09.109 19558831

[30] Li W Zhang Y Song Q Lan Y He HY Ma J. [Correlation Between Contrast-Enhanced Ultrasound and Risk of Tumor Recurrence in Papillary Thyroid Carcinoma]. Zhongguo Yi Xue Ke Xue Yuan Xue Bao (2021) 43(3):343–9. doi: 10.3881/j.issn.1000-503X.13828 34238409

[31] Wei X Li Y Zhang S Gao M. Prediction of Thyroid Extracapsular Extension With Cervical Lymph Node Metastases (ECE-LN) by CEUS and BRAF Expression in Papillary Thyroid Carcinoma. Tumour Biol (2014) 35(9):8559–64. doi: 10.1007/s13277-014-2119-2 24863948

[32] Zhang B Jiang YX Liu JB Yang M Dai Q Zhu QL. Utility of Contrast-Enhanced Ultrasound for Evaluation of Thyroid Nodules. Thyroid (2010) 20(1):51–7. doi: 10.1089/thy.2009.0045 20067379

[33] Yeh MW Bauer AJ Bernet VA Ferris RL Loevner LA Mandel SJ. American Thyroid Association Statement on Preoperative Imaging for Thyroid Cancer Surgery. Thyroid (2015) 25(1):3–14. doi: 10.1089/thy.2014.0096 25188202 PMC5248547

[34] Stefanova DI Bose A Ullmann TM Limberg JN Finnerty BM Zarnegar R. Does the ATA Risk Stratification Apply to Patients With Papillary Thyroid Microcarcinoma? World J Surg (2020) 44(2):452–60. doi: 10.1007/s00268-019-05215-4 31605172

